# FUNCTIONAL ASSESSMENT OF THE SHOULDER IN JIU-JITSU BLACK BELT ATHLETES

**DOI:** 10.1590/1413-785220233105e264796

**Published:** 2023-10-23

**Authors:** EWERTON BORGES DE SOUZA LIMA, JONATHAS TEIXEIRA SALLES, MATHEUS DE TOLEDO VENTURA, CARLOS VICENTE ANDREOLI, ALBERTO DE CASTRO POCHINI, PAULO SANTORO BELANGERO, BENNO EJNISMAN

**Affiliations:** 1 Universidade Federal de São Paulo, Escola Paulista de Medicina, Centro de Trauma Esportivo, São Paulo, SP, Brazil

**Keywords:** Shoulder, Pain, Athletic Injuries, Ombro, Dor, Lesões Esportivas

## Abstract

Objective: To assess the epidemiological profile of Jiu-Jitsu black belt athletes, including the prevalence of pain and shoulder function. Methods: Cross-sectional study carried out with Jiu-Jitsu athletes from 2014 to 2016. The studied variables were: sex, age, dominant limb, weight, height, profession, time of Jiu-Jitsu practice, weekly training hours, other practiced sports, comorbidities, injuries and previous surgeries, medications and habits. For the functional assessment of the shoulder, the ASES Score was used. Results: 53 male athletes were evaluated. There was a prevalence of alcohol consumption (60.4%) and supplement use (32.1%). The practice of other sports included weight training (49.1%) and other martial arts (17%). There was a prevalence of knee (66.0%) and shoulder (52.8%) injuries and, in some cases, the need for surgical procedures. There was a prevalence of shoulder pain (73.6%) and more than half of the athletes (52.9%) had minimal or moderate limitation of shoulder function. Conclusion: Jiu-jitsu black belt athletes often have a history of injuries, with the shoulder being the second most affected body part. In more than half of the athletes, there was a prevalence of shoulder pain and functional limitation, according to the ASES Score. *Level of evidence III, Retrospective comparative study.*

## RESUMO

Objetivo: Avaliar o perfil epidemiológico de atletas faixas-pretas de jiu-jitsu, incluindo a prevalência de dor e a função do ombro. Métodos: Estudo transversal realizado com atletas de jiu-jitsu entre 2014 e 2016. As variáveis estudadas foram: sexo, idade, membro dominante, peso, altura, profissão, tempo de prática do esporte, horas semanais de treino, outros esportes praticados, comorbidades, lesões e cirurgias prévias, medicamentos e hábitos. Para a avaliação funcional do ombro, foi utilizado o escore American Shoulder and Elbow Surgeons Standardized Shoulder Assessment Form (ASES). Resultados: Foram avaliados 53 atletas, todos do sexo masculino. Houve alta prevalência de consumo de bebida alcoólica (60,4%) e uso de suplementos (32,1%). A prática de outros esportes incluiu musculação (49,1%) e outras artes marciais (17%). Houve alta prevalência de lesões no joelho (66,0%) e no ombro (52,8%), e em alguns casos houve a necessidade de procedimentos cirúrgicos. Verificou-se alta prevalência de dor no ombro (73,6%), sendo que mais da metade dos atletas (52,9%) apresentaram limitação mínima ou moderada da função do ombro. Conclusão: Atletas faixas-pretas de jiu-jitsu frequentemente apresentam histórico de lesões, sendo o ombro o segundo local mais acometido. Houve alta prevalência de dor nos ombros e limitação funcional conforme o escore ASES em mais da metade dos atletas. *Nível de evidência III, Estudo comparativo retrospectivo*.

## INTRODUCTION

Combat sports are defined as the clash between two individuals with the goal of “finishing” the opponent through the fight. Brazilian Jiu-Jitsu (BJJ) is an important combat sport modality, which has been gaining popularity over the years.^1^ BJJ is a martial art rooted in Kodokan, the pre-war Judo, and has been undergoing modifications.^2^ ^), (^ ^3^ ^), (^ ^4^ It is known as the “gentle art” and its aim is to defeat the opponent through projections, chokes, twists and immobilizations resulting from tension force on the joints.^5^

The hierarchy of this modality is based on the system of belts, granted according to the athlete’s experience, skills, and time practicing the sport.^6^ In BJJ, the black belt represents an advanced level of technical and practical skills. To be eligible for a black belt, an athlete is required to be at least 19 years old and have spent at least one year as a brown belt. To progress from the black belt, the athlete must practice and teach at this level for at least three years.^7^ ^), (^ ^8^

BJJ is based on the use of force of the opponents against themselves, not including movements such as kicks against the opponent. Although less energy is put into the moves, BJJ athletes are subject to injuries, especially those with blue and black belts, mainly the latter. The objective of this study is to evaluate the epidemiological profile of black belt Jiu-Jitsu athletes and the prevalence of pain and shoulder function in this specific population.

## METHODS

This is a cross-sectional epidemiological study, conducted from 2014 to 2016, with black belt Jiu-Jitsu athletes, with at least 10 years of practice, in several training centers in the city of São Paulo. This study was approved by the Medical Ethics Committee of the Federal University of São Paulo under registration number 57674116.9.0000.5505.

### Data collection and sample

Black belt athletes with at least 10 years of Jiu-Jitsu practice were evaluated, composing a representative sample of a group of experienced and high-level athletes. The inclusion criteria were: Jiu-Jitsu athletes, black belt graduation, at least 10 years of practice, over 18 years of age. Exclusion criteria were: systemic disease with joint involvement and non-acceptance of the informed consent form (ICF).

Data collection was performed in gyms during training hours. The gyms were randomly selected within a 5 km radius of our research center. The data were collected by a single researcher, who visited the gyms on different days and times without giving athletes prior notice. After accepting the ICF, each athlete was given a previously tested self-administered questionnaire, with open and closed questions. The researcher was present during the completion of questionnaires to answer any questions. After completing the questionnaire, a physical evaluation was performed with the execution of special maneuvers for the shoulder and goniometry, performed with a manual goniometer.

### Evaluated variables

The demographic variables were: gender, age, dominant limb, weight, height, profession, time of Jiu-Jitsu practice, weekly training hours, other sports practiced, comorbidities, injuries and previous surgeries, medications and habits. For the functional evaluation of the shoulder, the American Shoulder and Elbow Surgeons Standardized Shoulder Assessment Form of the American Academy of Orthopaedic Surgeons (ASES/AAOS) was used. According to the score,^9^ ^), (^ ^10^ the athletes were classified as: 1) asymptomatic (> 93.9), 2) symptomatic without functional limitation (77.7 to 93.9), 3) minimal functional limitation (54.5 to 77.7), 4) moderate limitation (32.5 to 54.5).

On the physical examination, the evaluated variables were: passive and active range of motion (elevation, lateral rotation and medial rotation with abduction at 90°) and special maneuvers for evaluation of the rotator cuff (Jobe, Patte and Gerber tests), impact (Neer, Hawkins-Kennedy and Yokum maneuvers), biceps-labral injury (Speed, Yergason and O’Brien) and instability (apprehension and Fukuda). Elevations lower than 150°, lateral rotations lower than 60° and medial rotations lower than 50º^11^ were considered as deficit in the motion range.

### Statistical analysis

For the statistical analysis, SPSS V20, Minitab 16 and Excel Office 2010 software were used and a significance level of 5% (p<0,05) was defined. To evaluate the statistical dependence of two variables, the Chi-Square test was used; for two-by-two comparison of independent variables, the Mann-Whitney test was used; and for the analysis of the degree of association of two variables, Spearman’s correlation was used. Non-parametric tests were used due to the identification of a sample without a distribution of normality by the Kolmogorov-Smirnov test.

## RESULTS

### Demographic characteristics

The sample consisted of 53 athletes from seven different gyms. All athletes evaluated were male. The mean age was 34.6 years (min: 30, max: 45), the mean body mass index (BMI) was 26.34 (min: 22.73; max: 31.74), the average training time was 13.8 years (min: 10; max: 25), the average weekly training time was 10 hours (min: 3; max: 40).

### Life habits and sport practice

Forty-two athletes practiced another sport besides Jiu-Jitsu, especially weightlifting (49.1%) and other martial arts (17%). Thirteen athletes (24.5%) were professional Jiu-Jitsu athletes and the others practiced recreationally. Five (9.4%) athletes had some clinical comorbidity, including hypertension, type 2 diabetes, gastritis and asthma (Table 1).

**Table 1:** Prevalence of life habits, sports practice, injuries and surgeries in black belt Jiu-Jitsu athletes.

Habits	n	%
Anabolic steroid	9	17.0
Supplement	17	32.1
Alcoholism	32	60.4
Smoking	4	7.5
Sports		
Martial Art	9	17,0%
Football	4	7.5%
Weightlifting	26	49.1%
Cycling	4	7.5%
Surfing	7	13.2%
Crossfit	4	7.5%
Water Polo	1	1.9%
Skateboarding	2	3.8%
Swimming	1	1.9%
Jogging	4	7.5%
Volleyball	1	1.9%
Rugby	1	1.9%
Total	42	79.2%
Surgeries		
Shoulder	3	5.7 %
Hand and Fist	3	5.7 %
Knee	4	7.5%
Foot and Ankle	1	1.9%
Total	11	20.8%

Regarding life habits, nine (17%) athletes confirmed the use of anabolic steroids at some point in their sports lives, 17 (32.1%) used nutritional supplements, 32 (60.4%) reported drinking alcohol socially and 4 (7.5%) were smokers.

### Injuries and surgeries

**Figure 1:**
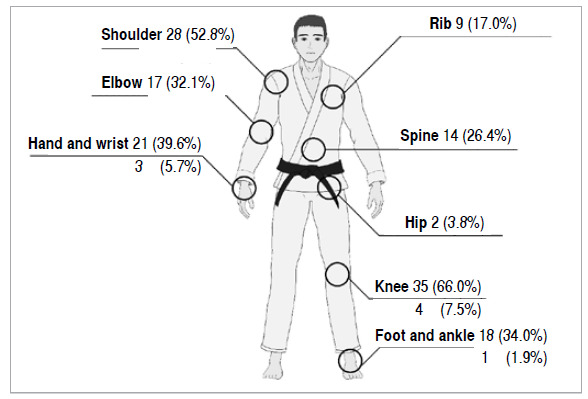
Prevalence of injuries and surgeries by body part. The values above the lines refer to the lesions and those below refer to the number of surgeries.

Forty-eight (90.6%) athletes had injuries during training or competitions, especially knee (66%) and shoulder (52.8%) injuries. Figure 1 shows the data on the prevalence of injuries in Jiu-Jitsu practitioners. Eleven (20.75%) athletes underwent surgical treatment for orthopedic injuries, including knee (7.55%), shoulder (5.66%), hand and wrist (5.66%), foot and ankle (1.89%) injuries.

### Shoulder evaluation

Thirty-nine (73.6%) athletes reported having shoulder pain. Range of motion (ROM) deficit was identified in 18 (34.0%) athletes. Loss of lateral rotation was the most prevalent (32.1%), followed by loss of elevation (7.6%) and medial rotation (5.7%) (Table 2). Patients with and without ROM deficit were not statistically different when compared in relation to quantitative variables (age, BMI, training time and weekly training hours). Decreased ROM showed a statistically significant relationship with positivity in special tests for biceps-labral injuries (p=0.042) and cuff injuries (p=0.004).

**Table 2:** Movements and elevation deficit, lateral rotation and medial rotation in black belt Jiu-Jitsu athletes.

	ELEVATION		LATERAL ROTATION		MEDIAL ROTATION	
	RIGHT	LEFT	RIGHT	LEFT	RIGHT	LEFT
**ACTIVE**	173°	174.8°	67.8°	69.2°	81°	83.2°
**PASSIVE**	174.6°	176.8°	70.5°	71.8°	82.5°	84.2°
**DEFICIT**	4 (7.6%)		17 (32.1%)		3 (5.7%)	

Only 26.4% of the athletes did not present positivity in the special tests. Nine (17.0%) wrestlers tested positive for one disease, 13 (24.5%) for two, 11 (20.8%) for three and six (11.3%) for four. Tests for Impingement Syndrome showed the highest positivity prevalence (54.7%), followed by tests for Rotator Cuff injuries (52.8%), biceps-labral injuries (39.6%) and instability (26.4%). There was no statistically significant relationship between the special tests and the quantitative variables, sports level, alcohol use, cigarettes or anabolic steroids.

The mean ASES score was 78.2 (min: 42; .max: 100), and 16 (30.2%) athletes presented scores compatible with normal shoulder function, 9 (17.0%) with some symptom without functional limitation, 25 (47.2%) with minimal limitation and 3 (5.7%) with moderate limitation. The ASES mean in patients with and without ROM deficit was 72.1 and 81.6, respectively, but without statistical difference (p=0.058). ASES had a statistically significant correlation with age (p=0.038): the higher the age, the lower the ASES and vice versa. Athletes with positivity in special tests presented worse ASES scores, and this association was significant for cuff tests (p=0.001) and for impact (p=0.003).

**Table 3:** Injuries and special tests for shoulder evaluation in black belt Jiu-Jitsu athletes.

Injury	Maneuver	Total	Right	Left	Dominant	Bilateral
**Rotator cuff**	Jobe	11 (20.75%)	3 (5.66%)	8 (15.09%)	5 (9.43%)	0
	Patte	21 (39.62%)	7 (13.20%)	15 (28.30%)	10 (18.87%)	1 (1.89%)
	Gerber	15 (28.30%)	6 (11.32%)	12 (22.64%)	8 (15.09%)	3 (5.66%)
	Total	28 (52.83%)	11 (20.75%)	22 (41.51%)	14 (26.42%)	5 (9.43%)
**Impingement Syndrome**	Neer	14 (9.43%)	3 (5.66%)	11 (20.75%)	5 (9.43%)	0
	Hawkins-Kennedy	13 (24.53%	10 (18.87%)	6 (11.32%)	11 (20.75%)	3 (5.66%)
	Yokum	15 (28.30%)	5 (9.43%)	12 (22.64%)	7 (13.21%)	2 (3.77%)
	Total	29 (54.72%)	13 (24.53%)	19 (35.84%)	14 (26.42%)	3 (5.66%)
**Biceps-Labral Injury**	Speed	4 (7.55%)	2 (3.77%)	2 (3.77%)	4 (7.55%)	0
	Yergason	5 (9.43%)	3 (5.66%)	2 (3.77%)	3 (5.66%)	0
	O'Brien	18 (33.96%)	12 (22.64%)	14 (26.41%)	13 (24.53%)	8 (15.09%)
	Total	21 (39.62%)	14 (26.41%)	15 (28.30%)	16 (30.19%)	8 (15.09%)
**Glenoumeral instability**	Apprehension	14 (9.43%)	7 (13.20%)	7 (13.20%)	6 (11.32%)	0
	Fukuda	1 (1.89%)	1 (1.89%)	1 (1.89%)	1 (1.89%)	1 (1.89%)
	Total	13 (24.53%)	9 (16.98%)	5 (9.43%)	5 (9.43%)	1 (1.89%)

## DISCUSSION

This study investigated the epidemiological profile, pain prevalence and shoulder function of black belt Jiu-Jitsu athletes. In the evaluated athletes, high prevalence of alcohol consumption; supplement use; practice of other sports; knee and shoulder injuries and, in some cases, the need for surgical procedures were observed. A high prevalence of shoulder pain was also observed, and more than half of the athletes had minimal or moderate shoulder function limitations.

The study highlights the specific black belt population, athletes who have high performance and long experience in the sport. Regarding the “gender” parameter, 100% (53) of athletes evaluated were male. Our sample was larger when compared to other studies conducted in Brazil.^12^ ^), (^ ^13^ It is important to highlight that, although our sample is predominantly male, in recent years, Jiu-Jitsu has aroused women’s interest, especially for the benefits tied to it.^12^

In the present study, there was a high prevalence of alcohol consumption. We identified only two studies in the literature that reported on the consumption of alcoholic beverages by Jiu-Jitsu practitioners.^14^ ^), (^ ^15^ One of the studies reported that 90% (n=9) of the athletes consumed alcoholic beverages twice a week.^14^ The other study indicated that 45.1% of the Jiu-Jitsu athletes consumed alcoholic beverages.^15^ It is important to consider that Jiu-Jitsu is a physical activity that takes great dedication, physical effort and continuous training. The ingestion of alcoholic beverages by athletes can delay reflex effects, making them slower when defending themselves from moves, which may result in a higher injury frequency.^15^

There was also a high supplement consumption by the athletes in the study. Another study conducted in Brazil also found a high prevalence of consumption of dietary supplements by Jiu-Jitsu practitioners.^16^ Food supplements can be used to improve performance in sports, but they should not be considered a conventional diet food and should be used under guidance. Athletes should also know the benefits and harms of overconsumption of these products.^16^

Most black belt Jiu-Jitsu athletes practiced other sports, especially weightlifting and martial arts. Although the practice of different sports is common, especially with weightlifting, we did not identify studies in the national and international literature that reported the practice of Jiu-Jitsu along other modalities and the increase or reduction of injury risk.

The topography of injury incidences in this sport is still divergent in the literature. Most research concludes that the highest incidence of injuries happens in the knees or hands, followed by the shoulders and elbows.^1^ ^), (^ ^5^ ^), (^ ^17^ According to Machado et al.^17^ most shoulder injuries occur in BJJ athletes when they apply or receive a fall or scraping move, and when they receive a move called “shoulder lock”. The injury mechanisms described are traction on the joint, a direct trauma of the shoulder region with the mat, with this limb abducted or adducted, which may be associated with an external rotation, when abducted, or a fall on the shoulder, resulting in a hyperextension.^17^

There was a high prevalence of knee and shoulder injuries and, in some cases, the need for surgical procedures. Our findings are in agreement with other studies published in Brazil that report on knee and shoulder injuries.^1^ ^), (^ ^12^ ^), (^ ^13^ ^), (^ ^18^ ^), (^ ^19^ ^), (^ ^20^ In addition, another study described that 15% of athletes required surgery after the injury.^4^ The incidence of injuries in Jiu-Jitsu can occur as in any other sport.^12^ Injuries, such as bruises, dislocations, fractures, sprains and nonspecific pain may occur during the training or competition phases, as a result of a program in which activities have been incorrectly planned or executed.^12^ ^), (^ ^21^

In the present study, there was also a high prevalence of shoulder pain (73.6%). This result is important, mainly because there is a lack of studies that address shoulder pain in Jiu-Jitsu athletes. This finding represents a wake-up call and injury prevention efforts should be considered. With epidemiological data it is possible to outline better preventive and treatment conducts, aiming at a more agile reinsertion of the athlete into sports practice without functional deficits.^1^

More than half of the athletes had minimal or moderate shoulder function limitation, and older athletes and positivity in rotator cuff and impact tests were associated with worse scores. We did not identify other studies that addressed shoulder function in Jiu-Jitsu athletes.

The strong point of this study is the inclusion of a specific population of black belt athletes; however, this limits the possibility of generalizing the results to other categories of BJJ practitioners. Another relevant aspect of this study is the evaluation of shoulder pain and function in Jiu-Jitsu athletes, in which there is a gap in the literature, both in national and international studies. The possible limitations of this study are related to the memory bias associated with the self-report of functional limitation in the shoulder evaluation by the ASES Score and the fact that the sample studied did not include female athletes. We highlight that the sample size is representative of this population, and these data are fundamental to stimulate the development of clinical treatments and prevent pain and function limitation in Jiu-Jitsu athletes.

## CONCLUSION

Black belt Jiu-Jitsu athletes often have a history of injuries, with the shoulder being the second most affected body part. Impingement Syndrome tests showed the highest prevalence of positivity, followed by Rotator Cuff injury tests. ROM deficit was common and is associated with positivity in tests for cuff and biceps-labral injuries. More than half of the athletes presented some degree of functional limitation by the ASES score, and both older athletes and positivity in cuff and impact tests were associated with worse scores.
